# Evaluating the impact of an educational intervention on the history of racism in America for teaching structural competency to medical academicians

**DOI:** 10.1186/s12909-024-05626-5

**Published:** 2024-06-07

**Authors:** Jason E. Glenn, Kristina M. Bridges, Kakra Boye-Doe, LesLee Taylor, Jill N. Peltzer, Shawn Leigh Alexander, Danielle Binion, Matthew Schuette, Carrie L. Francis, Jerrihlyn L. McGee

**Affiliations:** 1 Department of History and Philosophy of Medicine, University of Kansas Medical Center, Kansas City, KS USA; 2 Department of Family Medicine and Community Health, University of Kansas Medical Center, Kansas City, KS USA; 3 Department of Psychiatry, Northwestern University School of Medicine, Chicago, IL USA; 4 Department of Physical Therapy, Rehabilitation Science, and Athletic Training, University of Kansas Medical Center, Kansas City, KS USA; 5 School of Nursing, University of Kansas Medical Center, Kansas City, KS USA; 6 Department of African and African American Studies, University of Kansas, Lawrence, KS USA; 7 Office for Diversity, Equity and Inclusion, University of Kansas Medical Center, Kansas City, KS USA; 8 Director of Institutional Research and Academic Analytics, University of Kansas Medical Center, Kansas City, KS USA; 9 Otolaryngology-Head and Neck Surgery, University of Kansas Medical Center, Kansas City, KS USA

**Keywords:** Health iniquities, History of medicine, Systemic racism, Structural determinants of health, Structural competency, Continuing professional development

## Abstract

**Background:**

A challenge facing many Academic Health Centers (AHCs) attempting to revise health professions education to include the impact of racism as a social and structural determinant of health (SSDoH) is a lack of broad faculty expertise to reinforce and avoid undermining learning modules addressing this topic. To encourage an institutional culture that is in line with new anti-racism instruction, we developed a six-part educational series on the history of racism in America and its impact on contemporary health inequities for teaching structural competency to health professions academicians.

**Methods:**

We developed a six-hour elective continuing education (CE) series for faculty and staff with the following objectives: (1) describe and discuss race as a social construct; (2) describe and discuss the decolonization of the health sciences and health care; (3) describe and discuss the history of systemic racism and structural violence from a socio-ecological perspective; and (4) describe and discuss reconciliation and repair in biomedicine. The series was spread over a six-month period and each monthly lecture was followed one week later by an open discussion debriefing session. Attendees were assessed on their understanding of each objective before and after each series segment.

**Results:**

We found significant increases in knowledge and understanding of each objective as the series progressed. Attendees reported that the series helped them grapple with their discomfort in a constructive manner. Self-selected attendees were overwhelmingly women (81.8%), indicating a greater willingness to engage with this material than men.

**Conclusions:**

The series provides a model for AHCs looking to promote anti-racism and structural competency among their faculty and staff.

**Supplementary Information:**

The online version contains supplementary material available at 10.1186/s12909-024-05626-5.

## Background

The primary cause of health inequity in the United States is structural racism [1]. Stark inequities in morbidity and mortality among minoritized populations have persisted for generations. In 1899, W.E.B. Du Bois was the first to note that social conditions determined by the experience of racism and discrimination, not genetics, were the primary determinants of ill health of Black residents in Philadelphia, leading to their higher mortality rates [2]. The COVID-19 pandemic amplified and exacerbated the health inequities that have plagued marginalized communities for over a century [3–5]. The Black Lives Matter movement, brought to a crescendo with the brutal public murder of George Floyd, prompted many Academic Health Centers (AHCs) in the U.S., including associated schools of medicine, nursing and health professions, to increase their anti-racism efforts in the face of one of the most glaring examples of structural violence in America: the systemic killing of unarmed Black persons by police. These included efforts to eliminate bias from teaching materials and clinical algorithms, and mandated training in unconscious bias and other forms of discrimination [6]. Other academic institutions issued anti-racism statements or held community conversations to process the traumatic effect of structural racism [7]. Academic organizations were closely scrutinized for compliance with accreditation standards regarding diversity and many were found lacking [8]. 

Like many campuses, The University of Kansas Medical Center (KUMC) held community conversations and town hall meetings to discuss the legacies of racism at AHCs. University leadership instructed all departments to audit their curricula and remove instances of outdated race-based medicine and review their anti-racism instruction (including racism as a major structural determinant of health in the US). Initial efforts across the institution were disjointed, using non-standardized criteria to review curricula, and relying upon faculty with varying levels of content expertise in this area. To coordinate our efforts and improve implementation across the institution, KUMC adopted The REPAIR Project. **The REPAIR Project** (**REPA**iring **I**nstitutional **R**acism) is designed to address anti-Black racism and augment Black, Indigenous and People of Color (BIPOC) voices and presence in science and medicine. The project addresses racism in medicine and health professions as an educational problem by providing a theoretical framework for coordinating and implementing social justice and anti-racism curriculum throughout the medical center. The REPAIR framework began at the University of California at San Francisco [9] and is now being implemented at KUMC and Johns Hopkins School of Medicine as well.

The REPAIR framework is grounded in one question: How can academic health centers *repair* the harms caused by centuries of neglect, exploitation, and abuse in clinical encounters, and by biomedical systems of knowledge that have justified this mistreatment of persons of color by propagating and upholding theories of race, racial difference, and racial inferiority? The REPAIR framework explicitly recognizes that the European and American medical profession played a central role in giving *race* a *veneer* of biology, thus helping to naturalize the social hierarchies and health inequities that arose due to slavery and colonialism, by placing them in a discourse of biogenetic determinism.

We undertook the challenge of “repair” with the acknowledgement of social theory in Indigenous studies that troubles the concept of repair [10] in contexts where the material conditions for returning to a pre-harm status may no longer exist, especially in situations in which the harms committed led to losses of life. This acknowledgment includes insights from critical disability studies scholars who have long-critiqued distinct but interrelated concepts such as repair, rehabilitation, and cure [11, 12] in biomedical contexts. Each activity, training, and learning module developed under the REPAIR framework is designed to meet one or more of four pillars within the theoretical framework. These pillars also guide the development of new research, inform institutional policies and practices, and enhance community engagement. They are:

1. The **History** of the role of biomedicine in perpetuating racism and reinforcing theories of racial difference and racial inferiority.
2. **Decolonizing the health sciences** from bench to bedside, including deconstructing the use of race as a proxy in medical decision-making.
3. **Action**: developing strategies to address structural racism and other isms from a socio-ecological perspective.
4. **Accountability**: Envisioning how the field of biomedicine can **repair** these harms.

REPAIR Project initiatives at KUMC fall into four categories, each linked to one of the above pillars of understanding: curriculum development; faculty/staff educational development and training; clinical interventions to eliminate local health inequities; and community accountability.

As we began developing and rolling out new evidence-based curricular content around racism as a major structural determinant of health as part of REPAIR, initial student feedback revealed that it was not uniformly implemented by all instructors. Students reported that some preceptors lacked the expertise in structural competency [13] to adequately facilitate class discussions around the new topics, some undermined the new instruction by re-iterating some of the non-evidenced-based racial folklore [14] that permeates Western medicine, and some eschewed discussing the new content altogether.

To address these knowledge and attitudinal deficits in our faculty and staff, the KUMC Office for Diversity, Equity, and Inclusion (DEI) identified a team of content experts to develop an educational series designed to facilitate competency in understanding racism as *the* major social and structural determinant of health (SSDoH) in the United States. The series, open to all KUMC community members, explicated the roots of racism in Western thought and socio-political organization. It began by emphasizing the novelty of racialized human classification systems – a recent phenomenon in the 2.5 million years of human evolution that is barely 500 years old. Our series traced the colonial origins of racial thinking from its first emergence in the 16th century as a European religious *cum* natural philosophical system of classification demarcating *civilized* Christians from so-called *barbarous* pagans and infidels. From this emergence, the series followed the evolution of racial thinking as European and American intellectuals shrouded “race” in a *veneer of biology* in the 19th century, to lend racist prejudices, policies, and laws an air of scientific legitimacy. This biologization of racial human classificatory schemas transformed the idea of “race” into its current common understanding – the assumption that a few, specific *phenotypic features* (such as skin color, eye shape, hair texture, etc.) are good *proxies* for understanding innate *morphological and/or genetic differences* between groups of people so defined as *“races.”*

The series traced this development from the first Portuguese voyages into sub-Saharan Africa during the 1450s, then to the Indigenous-Columbian contact during the 1490s in the Caribbean which set in motion both the genocide of Native Americans and the importation of Africans as slaves. The series then mapped the evolution of race and racism during the transatlantic slave trade, the Civil War, Jim Crow era, and the Civil Rights Movement, culminating in a critical examination of how this legacy impacts health and wellbeing in contemporary contexts.

Though many academic institutions have implemented DEI programming and training over the past few years, published research looking at implementation and effectiveness is scarce [15]. This evaluative study of the series is meant to help fill the gap in implementation and effectiveness research pertaining to similar efforts. In this paper, we describe the educational program, explain the challenges to intervention and how they were overcome, report its outcomes, and offer suggestions for next steps in dismantling racism in academic health institutions.

## Methods

The idea for the series originated with KUMC’s Vice Chancellor for DEI and Chief Diversity Officer, co-author JM. Her office appointed an interdisciplinary team of faculty, students, and staff (all co-authors on this manuscript) with expertise in assessment, the History of Medicine, African and African American Studies, and the social and structural determinants of health to develop the series objectives, scope, and topic areas. The goal of the series was to deconstruct and denaturalize the idea of race as a proxy for bio-genetic difference, and elevate the understanding of *race* as a social construct and *racism* as one of the most powerful structural determinants of health in the U.S.

The team came up with four series objectives:

1. Contextualize the historical and systemic nature of racism and bias in the United States.
2. Describe the relationship between race and health as an outcome of the intergenerational impact of historic structural racism.
3. Explain the mechanisms through which structural racism shapes health outcomes using the socio-ecological framework.
4. Integrate lessons learned within each participant’s scope of influence.

The objectives for the series were largely driven by the perceived lack of structural competency among KUMC faculty and staff. This lack was identified by students seeking better training in these areas while frustrated by faculty who still taught race-based medicine; and as revealed during campus discussions that followed the murder of George Floyd outlining racism as *the* major structural determinant of health in the U.S.

The team then determined the critical topics to cover in each installment of the series, anticipating that most members of our community had never been taught a comprehensive history of racism and how integral it was to the founding of the Americas. As a committee, each member submitted a list of topics that they deemed important to cover in the series. We then voted on the best six topics to give a comprehensive overview of how integral racism has been to the history of the United States and its lasting impact on the contemporary health landscape, for an audience that we assumed had zero prior training.

Our top-voted series topics were:

1. The Birth of Race: The Genocide of Indigenous Peoples & Colonization of the Americas (delivered by co-author Glenn).
2. The Atlantic Slave Trade and the Evolution of Racial Thinking (delivered by co-author Glenn).
3. The Civil War (delivered by David Roediger [16]).
4. The Jim Crow Era (delivered by co-authors Bridges and Alexander).
5. The Civil Rights Movement (delivered by co-authors Bridges and Glenn).
6. The Modern Day – Contemporary Impacts of Historical Racism (delivered by co-authors Boye-Doe and Peltzer).

There were some topics proposed but that did not make our top list (e.g., History of Mass Incarceration, History of Racism in Policing, A History of Health Inequities, A History of Racism in Human Subjects Research, etc.), not because our committee didn’t share a sense of their vital importance, but because we felt our faculty and staff needed training in “the basics” – represented in our finalized topic list – before progressing to more advanced discussions that would rely on a shared foundation of those basics.

Our committee then determined which members had the content expertise to deliver each session, or if we would need to recruit from outside of our committee. Each lecture was created by the designated presenters. Each session was preceded by a ten-minute introduction designed to contextualize the history presented as to how it related to contemporary health inequities.

The series took place during the 2021–2022 academic year, in the middle of the COVID-19 pandemic, so organizers determined that all sessions would be delivered virtually over Zoom. Virtual delivery allowed for greater participation, automated data collection, and facilitated administering and collecting assessments. The ease and zero cost of recording each session over Zoom also facilitated further participation from persons who could not attend the live delivery of the content.

Virtual attendance over Zoom also meant that most participant data was automatically stored with names and emails, which then had to be de-identified. Prior to de-identification, co-author Glenn used the name and user email data (where present) to match to HR data to compose demographic profiles for participants, including preferred gender, race/ethnicity, administrator/faculty/staff/student/ or outside community member status, and departmental affiliation where appropriate. The data were then de-identified by removing names and user emails and assigning each participant a unique ID number. The de-identified data were then shared with co-author Bridges to perform demographic analysis while co-author Schuette performed statistical analysis on the de-identified assessment data.

Each of the six sessions in the series was accompanied by a pre-assessment. The pre-assessment asked each participant to rate their level of agreement with four statements: (1) “I can describe and discuss: race as a social construct versus race as a genetic factor”; (2) “I can describe and discuss: Decolonization of health sciences and health care”; (3) “I can describe and discuss: The history of systemic racism and structural violence from a socio-ecological perspective”; and (4) “I can describe and discuss: Reconciliation and repair in biomedicine.” Each statement was accompanied by a Likert scale of Strongly Disagree, Disagree, Neutral, Agree, or Strongly Agree. During the sixth and final session pre-assessment, an error occurred, and no attendees were asked question 4.

All attendees who registered and attended at least one session were emailed a post assessment following the final session, which asked participants to rate each of those questions again, followed by a three-question free-form survey: (1) “Compared to your initial expectations, how did the training align or change from those expectations?”; (2) “In what ways do you plan on implementing what you have learned?”; and (3) “What questions or concerns do you have following the Six-Part Educational Series?”

**Fig. 1:**
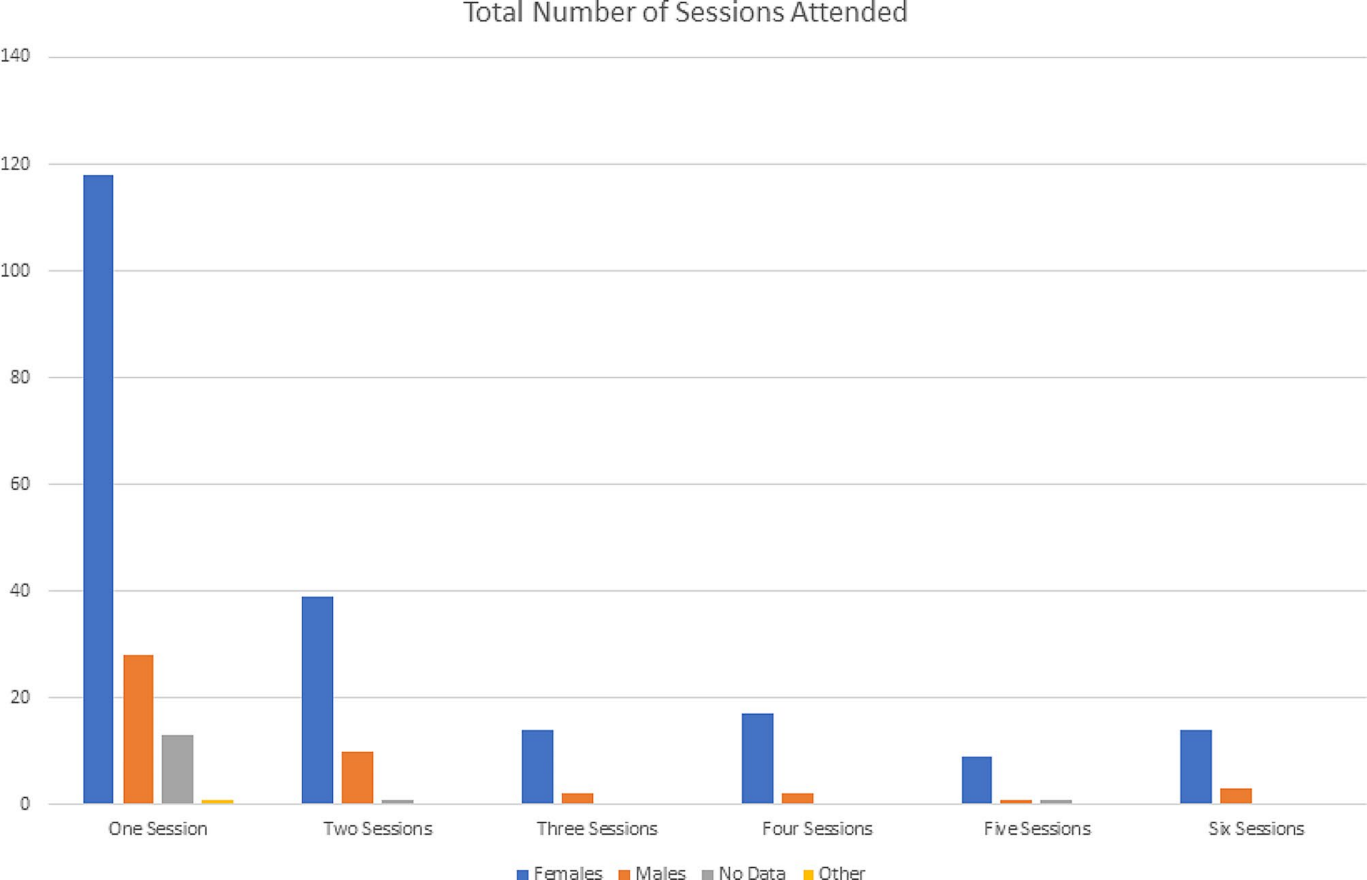
Number of sessions attended

**Fig. 2:**
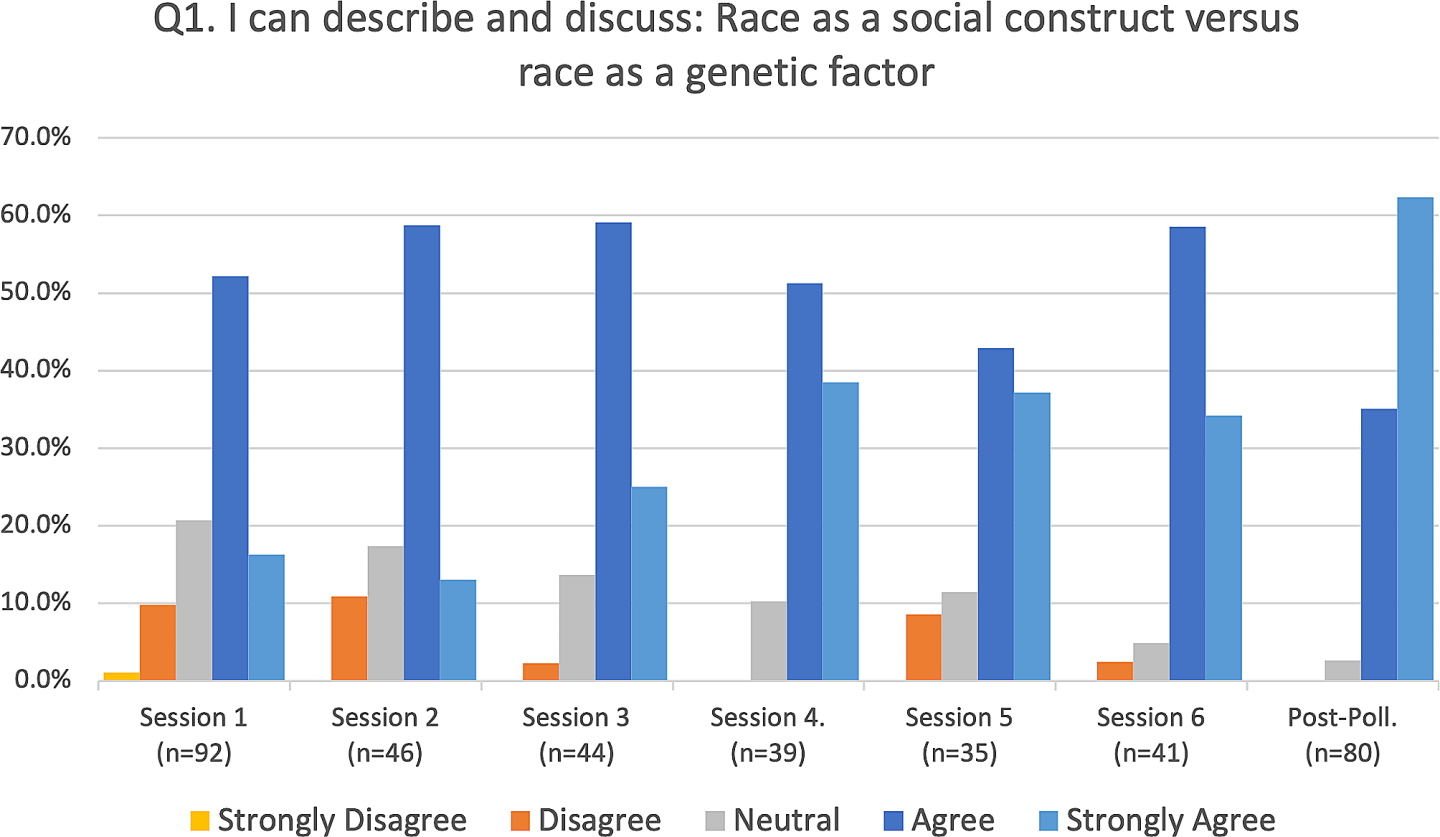
Race as a social construct versus race as a genetic factor

**Fig. 3:**
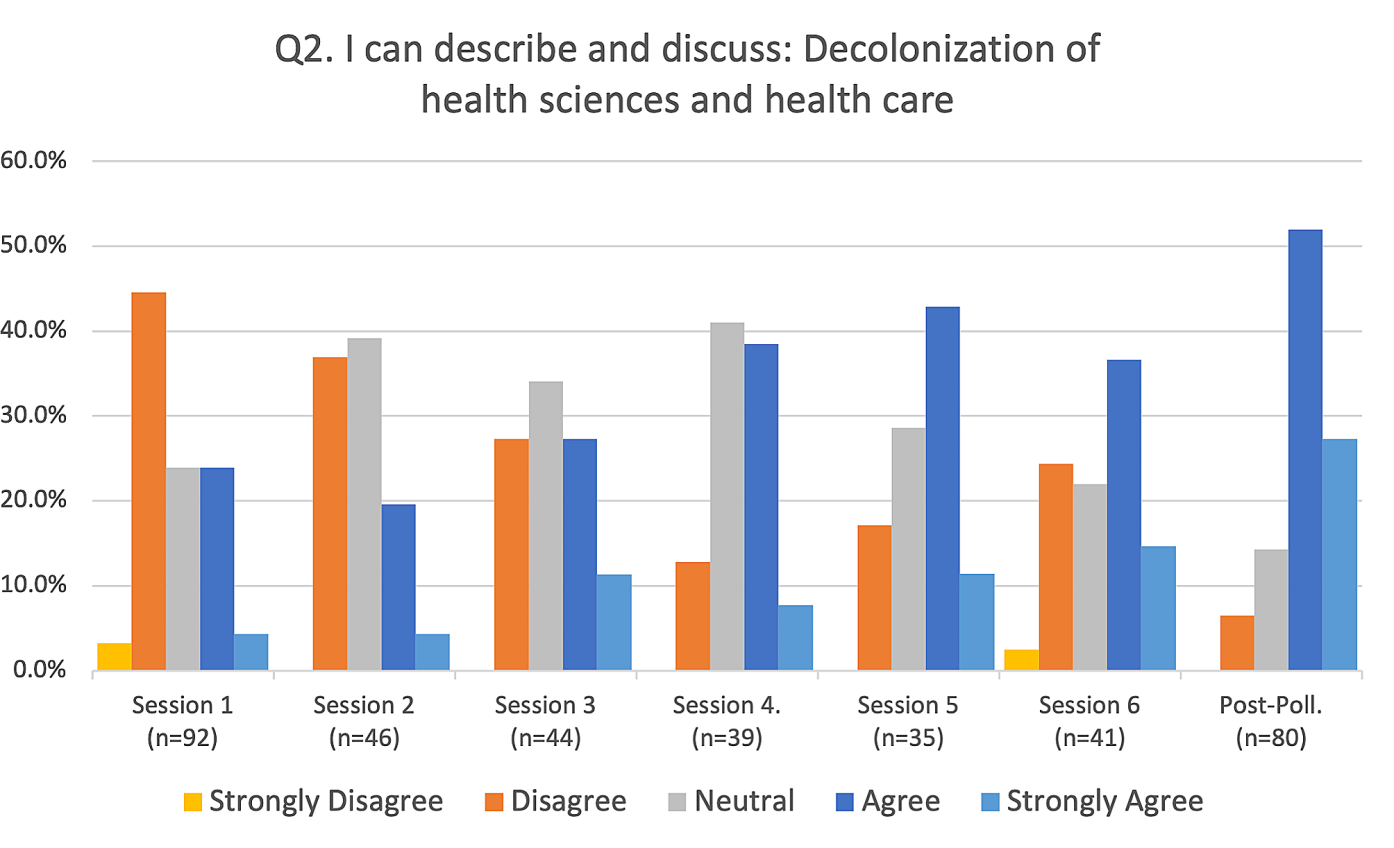
decolonization of the health sciences and health care

**Fig. 4:**
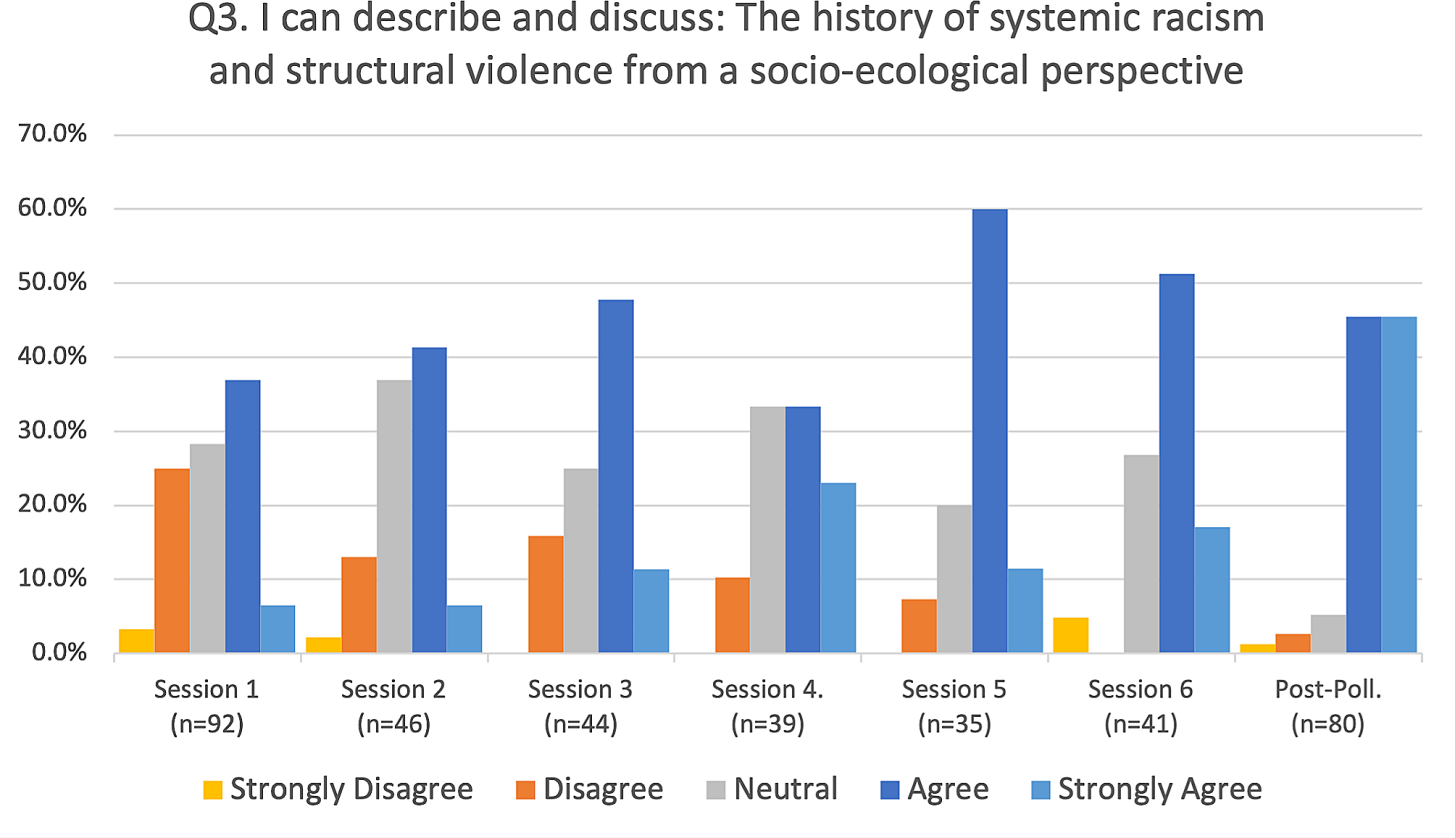
The history of systemic racism and structural violence

**Fig. 5:**
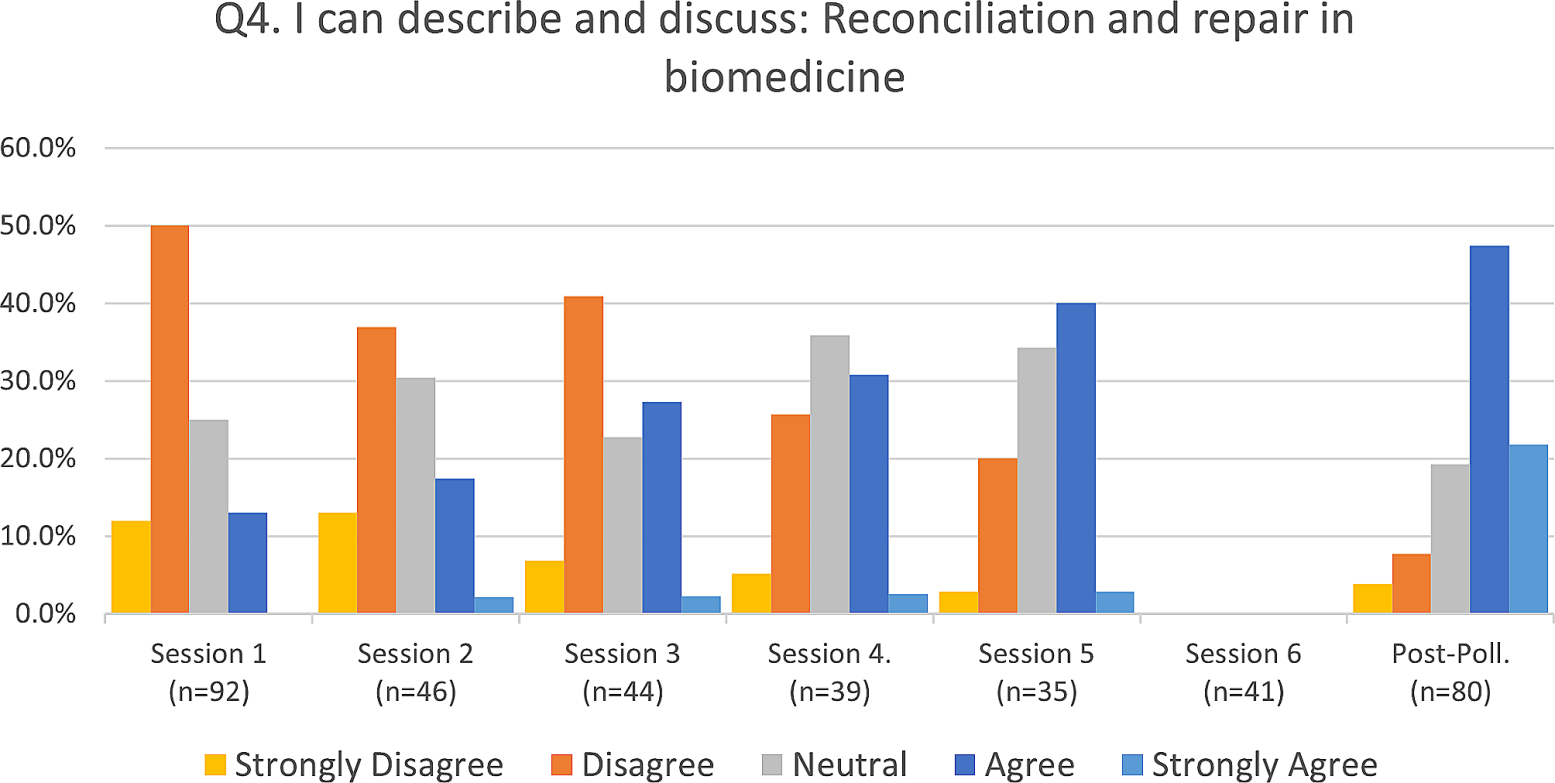
Reconciliation and repair in biomedicine

**Fig. 6:**
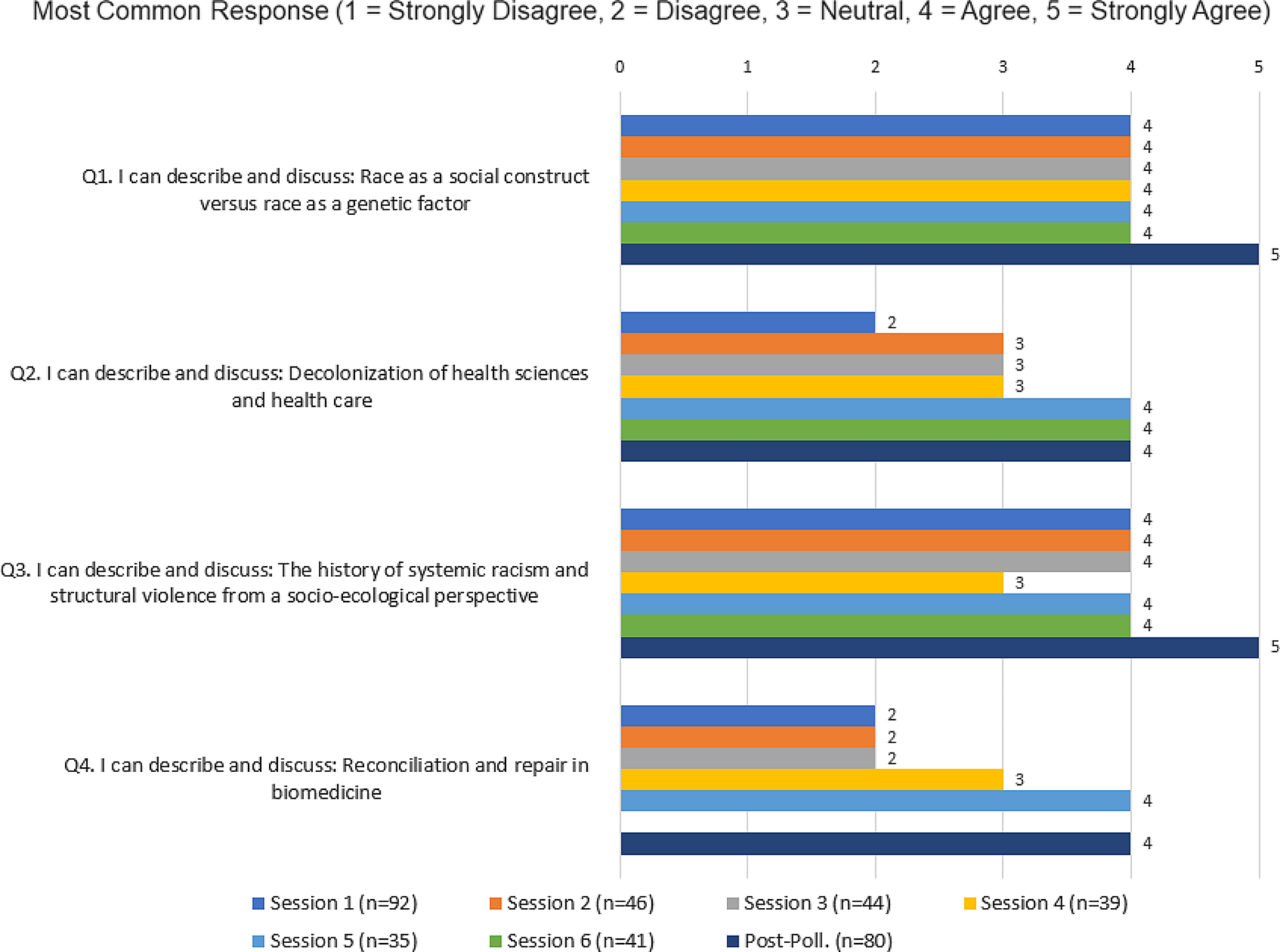
Most common session responses

### Statistical analysis

Responses to the four primary assessment questions were summarized (Figs. 2, 3 and 4, and 5) for each of the six sessions and the post-series poll, by calculating the percentage of respondents (vertical axis) who selected either strongly disagree, disagree, neutral, agree, or strongly agree, and representing this longitudinally (horizontal axis) from the first session to the post-series poll. The mode of the responses is shown as a clustered bar chart (Fig. 6), with the mode (horizontal axis) grouped by question and session date.

## Results

### Participants

The series enjoyed 273 unique participants who attended one or more sessions as they were delivered live, averaging 92 persons per session. A further 134 participants watched recorded versions of one or more sessions online. Of the persons who attended the live streaming, 160 people attended only one session, 50 people attended only two sessions, 16 people attended only three sessions, 19 people attended only four sessions, 11 people attended only five sessions, and 17 people attended all six sessions (Fig. 1). The average number of sessions attended was 2 out of 6. We are unable to determine the amount of overlap between persons who attended the sessions live and those who watched recorded versions.

Overwhelmingly, participants identified as female. Self-identified gender data was available for 258 participants, and of those, 211 (81.8%) identified as female, 46 (17.8%) identified as male, 1 (0.4%) identified as “other,” and there were 15 (5.8%) persons for whom no data was available (see Table 1).

There were 213 participants for whom racial/ethnic data could be collected. Of those, 133 (62.4%) identified as White; 38 (17.8%) identified as Black/African American; 20 (9.4%) identified as Asian [including 9 (4.2%) who identified as Indian, 6 (2.8%) Middle Eastern, and 5 (2.3%) Southeast Asians or Pacific Islanders]; 19 (8.9%) identified as Latinx/Hispanic; and 1 (0.5%) who identified as American Indian/Alaskan/Hawaiian Native). The gender gap skewing heavily toward women was present for every racial and ethnic group save for persons who identified as Middle Eastern, who were evenly split between males and females (Table 1).

The series drew attendees from across the health center and the surrounding community. Affiliation data was available for 210 (76.9%) participants. Of those, 59 (28%) were university administration/staff (including 2 members of executive leadership); 47 (22.4%) were clinical faculty; 37 (17.6%) were community members not affiliated with KUMC or any other AHC; 26 (12.4%) were research faculty; 14 (6.7%) were nurses; 27 (5.5%) were students (including 12 medical students, 6 (2.9%) post-docs, 6 (2.9%) graduate students; and 3 (1.4%) undergraduates) (Table 1).

**Table 1:** Demographics

Characteristic	Attendees *n* = 273
Gender, *n* (**% available data ***n*** = 258**)	
Female	211 (81.8)
Male	46 (17.8)
Non-binary	1 (0.3)
No data	15
Race/Ethnicity, *n* (**% available data ***n*** = 213**)	
White	133 (62.4)
Black	38 (17.8)
Hispanic	19 (8.9)
South Asian	9 (4.2)
Middle Eastern	6 (2.8)
No data	60
Role, *n* (**% available data ***n*** = 210**)	
University administrator/staff	59 (28.1)
Clinical faculty	47 (22.4)
Community member	37 (17.6)
Research faculty	26 (12.4)
Nurse	14 (6.7)
Medical student	12 (5.7)
Postdoctoral fellow	6 (2.9)
Graduate student	6 (2.9)
Undergraduate student	3 (1.3)
No data	63

The response rates for each of the six pre-assessments were 53.5%, 51.1%, 57.1%, 60.9%, 52.2%, and 51.3% respectively, with an average response rate of 54.4%. Out of the 273 attendees who were emailed and asked to complete a post-assessment, 80 did so, for a response rate of 29.3%. Our assessments revealed that attendees of this series demonstrated appreciable increased understanding of all four series objectives over the course of the series. Out of the four objectives, the first (“I can describe and discuss race as a social construct”) had the highest pre-series understanding, with 68.5% of respondents indicating that they either agreed or strongly agreed at session 1 and only 10.9% reporting that they disagreed or strongly disagreed (see Fig. 2). By contrast, the fourth objective for the series, (“I can describe and discuss: reconciliation and repair in biomedicine”) had the lowest pre-series understanding, with 62% of respondents reporting that they either disagreed or strongly disagreed at session 1 while only 13% reported that they agreed or strongly agreed (see Fig. 5).

Overall, objectives two (“I can describe and discuss: Decolonization of the Health Sciences and health care”) and four (“I can describe and discuss: reconciliation and repair in biomedicine”) had more participants who were unfamiliar with the concepts pre-series than who were familiar (see Figs. 3 and 5, and 6). For objective two, 47.8% of respondents indicated that they either disagreed or strongly disagreed at session one. This percentage was reduced to just 6.5% at the post-series poll following session six. We saw a similar degree of reduction in disagreement with objective four, from 62% at session 1 down to only 11.5% at the post-series poll following session six.

For objective three, “I can describe and discuss: The history of systemic racism and structural violence from a socio-ecological perspective,” most respondents (65.3%) polled before session one expressed either neutral or slight agreement, while only 6.5% of respondents expressed strong agreement. However, by the end of session six, strong agreement responses rose to 45.5% (see Fig. 4).

Participants responded to three optional open-ended questions during the post-series poll (Table 2). Many participants commented on the depth of the content delivered and the unexpected value of learning history to inform healthcare. Many other attendees commented on how the series helped them grapple with their discomfort around the topic of racism in a constructive manner. For some participants, the series sparked a plan to share what they learned with others and to change their behavior. Still, some patients had questions about how they could directly take action against racism.

**Table 2:** Post-series open-ended feedback

Question	Participant Responses
“**Compared to your initial expectations, how did the training align or change from those expectations?”**	“This program dove far deeper into the details of America’s history than I initially expected. Fantastic for learning where our country came from and how that factors into the policies and themes we see today.” “historical context of how deep and long racism is… beyond Civil War and US origins.” “it was more of a history lesson - cool! I guess you need to know how we got here to understand what needs to be done now.” “I did not expect to go so far back in history, but I am very glad [the presenters] did because it helped me better understand the foundation racism was built upon.” “It exceeded my expectations! I expected the content to make me uncomfortable and it did! … As it should have as a White, Catholic Christian individual.” “This has been one of the most impactful series I have experienced. Being educated on history that should have been taught in K-12, or at least university level has change [sic] how I view everyday events and interactions. I no longer blindly accept what I was taught about historical events in the past. I feel angry and I think that is a good place to start as I reevaluate what I believed was ‘truth’.”
**“In what ways do you plan on implementing what you have learned?”**	“I plan on speaking up when I see institutional fascism going on. I also plan on passing on the info to colleagues if the opportunity arises. And to read more on it.” “I’m going to share what I learned with others, so I get used to talking about the concepts.” “My family and I discuss topics like this on our weekly Zoom chats, and I plan to bring it up this Sunday. I never knew what ‘race as a social construct’ meant, and I do now.” “This has been one of the most impactful series I have experienced… Please make the recordings available to the public. I would like to share these with my family, kids, and community.” “I will look for ways that my worldview and beliefs shape how I think and what I think I am entitled to. I will use this as I try to understand how other people can believe and think differently from me.” “Taking into consideration that patients have lived a whole life before they had to come to the hospital. Everything a patient has been through has affected where they are now. Just being more aware of that. So that I can try to help moving forward in their healthcare journey.”
“**What questions or concerns do you have following the Six-Part Educational Series?**	“How do you start relations as a White person with people who have been annihilated for so long?” “What actions can individuals take to address structural racism when we see it? Include workplace and social organizations.”

## Discussion

Our pre- and post-polling data suggest that attendees found the series to be a powerful and rich learning experience. Complex concepts – such as race as a social construct, structural violence, decolonization, reconciliation and repair in biomedicine – all saw major advancements in participant understanding by the end of the series. A series of humanities and social science lectures were delivered to an audience trained largely in the biomedical sciences in a way that kept their attention and sustained their engagement over the course of six months.

Generally, female employees of KUMC outnumber males by a ratio of 3:2. However, female series attendees outnumbered males by a ratio of almost 5:1. Employees of the health center are also approximately 72% White. This means that both women and Black, Indigenous and People of Color (BIPOC) individuals (*n* = 80 or 37.6%) were over-represented as series attendees, though women were much more so. This is consistent with a 2022 Women in the Workplace Report which found that women are leading the charge for a more inclusive workplace and are two times as likely to invest time and energy in DEI activities, compared to their male counterparts [17]. White men accounted for only 9% of total attendees. Compared to their numbers on campus, they were the most underrepresented of any gender/racial group. Given that attendance was voluntary, this may indicate a general disinterest among White males in DEI educational programming, and a need to conduct greater targeted outreach for that population.

This also suggests that part of our success with this series likely benefits from some selection bias based on who chose to attend. While the marked improvement on all knowledge objectives among attendees over the course of the series was promising, these results may be boosted by the general amenableness to DEI concepts and activities by a predominantly female audience. Further research is needed to test whether persons who are more skeptical or hostile to DEI concepts and activities would progress as much in their learning if attendance to such a series was mandatory.

Though the series was primarily targeted to clinical and research faculty, administrative staff were our largest group of participants at 28%. This may be a function of the time that each installment of the series was delivered – over lunch – given that administrators and staff may be more likely to consistently have unstructured time during the noon hour. We do not necessarily see this as a shortcoming – all employees have a role to play in creating an anti-racist environment within an AHC – but it does suggest the need for special targeted programming when we specifically want to reach clinical faculty, researchers, or students.

The number of participants who were pleasantly surprised at the historical nature of the series is an indication of how integral history of medicine scholarship is to the development of an anti-racist and structurally competent biomedical workforce (when it doesn’t neglect the central role that racism and the concept of race played in the production of biomedical knowledge). It also demonstrates that there is an appetite for more of this type of learning on a medical campus. Unfortunately, it is also a commentary on how often teaching the history of medicine to students and faculty gets neglected in many AHCs [18]. 

All of our survey responses were collected anonymously, which limited our ability to measure any correlations between competency and the number of sessions attended, or demographic profile. We felt that collecting the assessment data anonymously was important for responders to feel safe in answering each question honestly and candidly. However, one institutional goal of this educational intervention is to assess whether or not direct care staff who participate and have improved competency in the series objectives will demonstrate more equitable treatment of the patients with whom they interact. Collecting the survey data anonymously makes this goal unobtainable. To correct this limitation in future educational interventions, we hope to devise an improved data collection system that deidentifies survey data while preserving its linkages to other participant data.

To capitalize off of the success of the intervention, we organized a follow-up series on six additional topics during the 2022–2023 academic year. For this follow-up series, we revisited many of the topics that were proposed for the first series but were ultimately set aside in favor first establishing a shared foundation of the basics. The topics covered in the second series were:

1. From Slavery to Mass Incarceration.
2. Race Throughout the Americas: Contrasting the Racial Cosmogonies of the U.S. and Latin America.
3. The Eugenics Movement, Genetic Determinism, and Blaming Victims: Understanding the American Desire to Attribute Health Inequities to Genetics.
4. Black Business Ownership, Communities, and Health.
5. Life as an African American student at the University of Kansas.
6. Discrimination, Stress and Allostatic Load: Understanding the Health Impacts of the Lived Experience of Racism.

The impact of this follow-up series is still being analyzed and will be discussed in a future publication. Our office of DEI is also partnering with the REACH Healthcare Foundation to develop updated and enhanced versions of each session in the series to offer it as continuing education and training to the foundation’s community partners and funding recipients. The two seasons from the series have been made available on open-source platforms for perpetual viewing to ensure accessibility and longevity.

In addition to increasing the overall institutional competency around our learning objectives, we were most interested in what plans participants have for implementing what they learned during the series. An obvious next step for us in future iterations of this curriculum is to target more clinicians and nurses, then track whether participation in this curriculum leads to improvements in satisfaction and health outcomes for their patients. The clinical intervention arm of KUMC’s REPAIR Project aims to do just that with the creation of health equity accountability dashboards for all clinical departments within the health system. Leveraging the past five years of EHR data, we are currently establishing health equity baseline dashboard scores for clinical departments and providers. We will then use future EMR data to track improvements, compared to the baseline score, for any clinician who has attended any DEI programming or training.

## Conclusions

The series provides a model for AHCs looking to promote anti-racism and structural competency among their faculty and staff, even in a conservative and politically contentious environment. Through interdisciplinary cross-campus collaborations and the use of a virtual platform, the series was developed, delivered, and assessed without needing to fundraise and without incurring any additional costs to the institution.

### Electronic supplementary material

Below is the link to the electronic supplementary material.

Supplementary Material 1

## Data Availability

The datasets used and/or analyzed during the current study are available from the corresponding author on reasonable request.

## Abbreviations

AHC: Academic Health Center
BIPOC: Black, Indigenous and People of Color
CE: Continuing Education
DEI: Diversity, Equity, and Inclusion
EMR: Electronic Medical Record
ID: Identification
KUMC: University of Kansas Medical Center
SSDoH: Social and Structural Determinants of Health
