# Supplementary material for: Evaluating the impact of an educational intervention on the history of racism in America for teaching structural competency to medical academicians
Source: BMC Med Educ. 2024 Jun 7;24:638. doi: 10.1186/s12909-024-05626-5 (PMC11157923; doi:10.1186/s12909-024-05626-5)
Supplement: Supplementary file 1 — Supplementary Material 1 [file 12909_2024_5626_MOESM1_ESM.docx]

**Evaluating the Impact of an Educational Intervention on the History of Racism in America for Teaching Structural Competency to Medical Academicians.**

**Appendix**

Online Recorded Versions of the Educational Series:

<https://www.youtube.com/watch?v=4JOXxm6jYug&list=PLb8-ONa2xkb3wXcDkLKFhdKN8x0rkI2cy>
